# Exposure to Microbial Metabolite Butyrate Prolongs the Survival Time and Changes the Growth Pattern of Human Papillomavirus 16 E6/E7-Immortalized Keratinocytes *in Vivo*

**DOI:** 10.1016/j.ajpath.2021.06.005

**Published:** 2021-10

**Authors:** Mengtao Li, Eva M. McGhee, Lauryn Shinno, Kellie Lee, Yi-Ling Lin

**Affiliations:** ∗ Division of Diagnostic and Surgical Sciences, School of Dentistry, University of California, Los Angeles, California; ‡ Gene Regulation Program, Jonsson Comprehensive Cancer Center, University of California, Los Angeles, California; † Department of Internal Medicine, School of Medicine, Charles R. Drew University of Medicine and Science, Los Angeles, California

## Abstract

Human papillomavirus (HPV) is a ubiquitous human pathogen that can be cleared by host immunity. Nonetheless, a small percentage of the patients develop persistent infection with oncogenic HPV, which poses an increased risk of developing HPV-associated malignancy. Although cell-mediated immunity is a known systemic factor, local factors that influence persistent HPV infection have not been fully investigated. HPV-related head/neck cancers have a strong site preference for the oropharynx, suggesting the existence of unique local factors that promote HPV-induced oncogenesis. The human oropharynx often harbors anaerobic bacteria that produce a variety of byproducts, including butyrate. Because butyrate is a potent epigenetic modulator, it could be an environmental factor influencing the development of HPV-positive oropharyngeal malignancy. In this study, we showed that butyrate treatment changed the property of HPV16 E6/E7-immortalized keratinocytes. *In vitro*, the treatment increased the cells' migration ability, slowed the growth, and increased the genotoxic resistance. When implanted in the syngeneic mice, the treated keratinocytes survived longer and exhibited a different growth pattern. The survival advantage obtained after butyrate exposure may increase the susceptibility of HPV-infected oropharyngeal keratinocytes to further malignant transformation. These results suggest that fermentation products of tonsillar bacteria may play an important role in the long-term persistence of high-risk HPV infection, which is a critical risk factor for developing HPV-positive oropharyngeal malignancy.

Human papillomavirus (HPV) is a small nonenveloped circular double-stranded DNA virus that infects keratinocytes. Among the five genera identified so far, α-papillomavirus is the most commonly studied group because of its association with cancer/precancerous lesions,^1^ and is subdivided into low-risk (eg, HPV6 and HPV11) and high-risk (eg, HPV16 and HPV18) genotypes. High-risk HPV encodes two major oncoproteins, E6 and E7, that play a pivotal role in driving the infected keratinocytes toward malignancy. The E6 oncoprotein inactivates the tumor suppressor and cell cycle checkpoint protein TP53, whereas the E7 oncoprotein inactivates the retinoblastoma (RB1) tumor suppressor protein.^2^^,^^3^ Together, E6/E7 oncoproteins target diverse cellular pathways involving cell cycle, apoptosis, and cell polarity, resulting in cell cycle deregulation and genome instability.^4^^,^^5^ Knockdown of *E6/E7* induces cell senescence or apoptosis, highlighting their crucial roles in the persistence of HPV-mediated cancers.^6^^,^^7^ Infection with high-risk HPV is responsible for ≈5% of all human cancers and accounts for 70% of oropharyngeal malignancies.^8^

HPV infection has caused an epidemic increase in the incidence of oropharyngeal cancers worldwide.^9^ HPV-related head and neck squamous cell carcinomas (SCCs) occur almost exclusively in the oropharynx.^10^^,^^11^ HPV-positive oropharyngeal SCC (OPSCC) often exhibits a high-grade morphology, nonkeratinized and basaloid, deviating from the conventional well-differentiated, keratinized SCC in the oral cavity.^12^ HPV-positive OPSCC appears to be molecularly and pathologically distinctive from its HPV-negative counterpart. Clinically, HPV-positive OPSCC has a unique pattern of metastasis, a longer disease-free interval, and a better prognosis.^13^ A new cancer staging system for HPV-positive OPSCC was established to guide treatment,^14^ partly because de-escalation of treatment does not compromise the cure rate but decreases morbidity caused by adverse effects of the therapy. However, treatment de-escalation for HPV-positive OPSCC has been recently discouraged after the publication of RTOG 1016 and De-ESCALaTE studies, both showing that outcomes of HPV-positive OPSCC patients strongly depend on the type of treatment received.^15^^,^^16^

HPV-related premalignant progression is traditionally studied on uterine cervical premalignant lesions. The derived cell lines have been the major tools for researchers to understand the biological process of HPV-associated malignant transformation.^17^ After prolonged passaging, the cell lines are able to undergo phenotypic progression from low-grade through high-grade dysplasia to invasiveness when differentiating into stratified squamous epithelium in organotypic raft epithelial tissue cultures.^18^^,^^19^ Unlike cervical cancers, the process involving HPV-positive oropharyngeal malignancy is less well understood, and at least two major issues remain to be elucidated. First, premalignant lesions (dysplasia) have rarely, if ever, been identified in clinical oropharyngeal specimens in the absence of an invasive component.^20^ Because premalignant lesions are present in HPV-related SCCs involving other anatomic sites, including the adjacent oral cavity,^21^ their absence in the oropharynx remains an enigma. Second, HPV-related head and neck cancers have a strong site preference for the oropharynx.^10^^,^^22^

The unusually high incidence of HPV-positive SCC in the oropharynx suggests the existence of unique local factors that promote HPV-induced oncogenesis. The oropharynx contains palatine tonsils and accessory lymphoid tissues of the Waldeyer ring that form deep crypts lined by patches of nonkeratinized stratified squamous epithelium and reticulated spongiotic epithelium with a gapped, noncontinuous basement membrane.^23^ The crypt epithelium is constantly infiltrated by lymphoid cells and forms a loose network abutting the subjacent lymphoid tissue. The deep crypts provide a low-oxygen habitat for anaerobic bacteria,^24^^,^^25^ which produce a variety of fermentation products, including butyrate, which contributes to halitosis.^26^ Butyrate is a histone deacetylase (HDAC) inhibitor and has been shown to function as an epigenetic modulator to condition intestinal immunity and improve the health of intestinal epithelium.^27^^,^^28^ However, butyrate has both protumor and anti-tumor properties that have been observed on cultured HPV-immortalized epithelial cells.^29^^,^^30^

Although HPV is a ubiquitous human pathogen, host immunity is capable of clearing the infection in most cases, including those caused by the high-risk group. However, a small percentage of the patients develop persistent infection, which is associated with an increased risk of cancer development.^31^, ^32^, ^33^ Although humans are the natural host of HPV, various studies have shown that HPV16 oncoproteins can target mouse TP53 and RB1 pathways similar to those in the human cells.^34^ Therefore, mice provide a valuable animal model to investigate HPV precancer-host or cancer-host interaction.

The current study showed that butyrate treatment changed the *in vitro* and *in vivo* properties of HPV16 E6/E7-expressing keratinocytes. The treatment prolonged the survival time and changed the growth pattern of the cells after injection into syngeneic, immunocompetent mice. The results suggest a link between the byproducts produced by tonsillar bacteria and chronic HPV infection.

## Materials and Methods

### Animal Use

C3H/HeNCrl mice were obtained from Charles River (Hollister, CA), and nonobese diabetic–severe combined immunodeficiency IL-2Rγnull mice were obtained from Radiation Oncology, University of California, Los Angeles (UCLA) (originally from Jackson Laboratory, Bar Harbor, ME). Mice were maintained under the care of the UCLA Division of Laboratory Animal Medicine. All animal studies were approved by the UCLA Institutional Animal Care and Use Committee and performed following the *Guide for the Care and Use of Laboratory Animals*.^35^ Mice were housed under controlled conditions (12-hour light/dark cycle), given water and food ad libitum, and monitored daily by trained staff. Both male and female mice, aged 2 to 4 months, were used in the study. The mice received a one-time injection of the immortalized keratinocytes (see below) [5 × 10^6^ cells in 50 μL phosphate-buffered saline (PBS)] into the masseter muscle under inhalation anesthesia (2% isoflurane). Mice were euthanized on the indicated days by CO_2_ inhalation, and the heads were harvested for histologic analysis.

### Retroviral Plasmids

pBABE-puro-hTERT (plasmid number 1771; a gift from Bob Weinberg, Massachusetts Institute of Technology, Boston, MA)^36^ and pBABE puro H-Ras V12 (plasmid number 9051; a gift from William Hahn, Dana-Farber Cancer Institute, Boston, MA) were purchased from Addgene (Watertown, MA). pMHPV16d was a gift from J. A. DiPaolo (National Institutes of Health, Bethesda, MD), which contains a head-to-tail dimer of HPV16 DNA.^37^ pMSCVneo, pMSCVhygro, and pMSCVpuro were purchased from Takara Bio (Mountain View, CA). *H-Ras V12* gene was released from pBABE-puro-H-Ras V12 plasmid with BamHI and SalI digestion, and ligated to pMSCVhygro vector cut with BglII and XhoI to establish pMSCVhygro-HRasV12. The *hTERT* gene was released from pBabe-puro-hTERT plasmid with EcoRI and SalI digestion, and subsequently cloned into pMSCVhygro vector cut with EcoRI and XhoI to establish pMSCVneo-hTERT. HPV16 *E6E7* fragment was PCR amplified from pMHPV16d with forward primers: 5′-GATCGTCGACATGCACCAAAAGAGAACT-3′ and reverse 5′-CTAGAAGCTTTTATGGTTTCTGAGAACA-3′. The *E6E7* gene was cloned into pSP72 plasmid (SalI and HindIII sites) and sequenced to ensure the accuracy of the sequence. The *E6E7* was released with SalI and XhoI digestion and then subcloned into pMSCVpuro (SalI and XhoI) to establish pMSCVpuro-HPV16 E6/E7. The 293FT cells were transfected with the MSCV retroviral vectors to produce retroviruses for cell infection, as previously described.^38^

### Establishment of Cell Lines

The base of the tongue was dissected from newborn mice and used as the source material to establish the keratinocyte cell lines. The harvested tissue was minced in PBS and digested in 0.64 mg/mL collagenase type 2 (number LS004174; Worthington Biochemical Corp., Lakewood, NJ) in PBS containing 0.01% trypsin and 0.1 mmol/L EDTA for 30 minutes in a 37°C shaker. An equal volume of RPMI 1640 medium with 10% fetal bovine serum (FBS) was then added to stop the digestion. The cells were collected by centrifugation, resuspended in Keratinocyte SFM (number 17005042; ThermoFisher, Waltham, MA), and plated on collagen-coated plates. After the formation of colonies, the cell medium was switched to keratinocyte media [1:1 mixture of Keratinocyte SFM and DF-K (a 1:1 mixture of calcium-free Dulbecco’s modified Eagle’s medium and Ham F12)] to support higher-density cell growth.^39^ The primary keratinocytes were immortalized by transduction with pMSCVneo-hTERT or pMSCVpuro-HPV16 E6/E7 retrovirus to establish the mouse oropharyngeal keratinocyte cell lines MOKT1 or MOKE6E7, respectively. MOKE6E7 was gradually adapted to and maintained in the keratinocyte media containing 5% FBS to select the cells with undifferentiated phenotype because FBS promotes keratinocyte differentiation.^40^ The two cell lines were further retrovirally infected with pMSCVhygro-HRasV12, which generated MOKT1/R and MOKE6E7/R cell lines. MOKE6E7/R was also maintained in the keratinocyte media containing 5% FBS. MOKE6E7 passage 15 was treated with 20 mmol/L butyrate for 48 hours to establish MOKE6E7_B_ passage 16 (the passage number assigned for MOKE6E7_B_ is continuous with that of MOKE6E7). MOKE6E7_B_ was further adapted to RPMI 1640 medium/10% FBS. RPMI 1640 medium contains 0.25 mmol/L calcium, which also promotes keratinocyte differentiation,^40^^,^^41^ and was used to enrich the cells with undifferentiated phenotype. To minimize genotype drifting, cell lines used in the studies were within passage 28 to 60. For *in vivo* experiments, MOKE6E7 and MOKE6E7/R were cultured in the keratinocyte media with 5% FBS, and MOKE6E7_B_ was cultured in RPMI 1640 medium with 10% FBS. For *in vitro* experiments, to maintain the same culture condition with MOKT1-derived cells, all MOKE6E7-derived cell lines were cultured in the keratinocyte media without FBS for 2 days before the experiment.

### Antibodies

The antibodies used in the study are summarized as follows: CD4 (clone 4SM95; number 14-9766-82), CD8 (clone 4SM15; number 14-0808-82), CD49b/Integrin Subunit Alpha 2 (ITGA2) (clone DX5; number 14-5971-85), forkhead box P3 **(**FOXP3) (clone FJK-16s; number 14-5773-82), inducible nitric oxide synthase/nitric oxide synthase 2 (number PA1-036), arginase 1 (ARG1; number PA5-29645), pan-cytokeratin (number PA1-27114), CD68 (clone FA-11; number 14-0681-82), and F4/80 (clone BM8; number 14-4801-82) were purchased from ThermoFisher Scientific (Waltham, MA). TP53 (clone POE316A/E9; number MABE1808) and tubulin (number T9026) were from MilliporeSigma (Burlington, MA). HPV16 E6 (number orb10837) for Western blot analysis and HPV16 E7 (number orb10573) for immunofluorescence were from Biorbyt LLC (St. Louis, MO). HPV16 E6 (clone C1P5; number NB100-2729) for immunofluorescence, HPV16 E7 (clone TVG 701Y; number NB100-2768) for Western blot analysis, and bromodeoxyuridine (BrdU; clone BU1/75; number NB500169) were from Novus Biologicals (Centennial, CO).

### Immunofluorescence

For pan-cytokeratin, TP53, HPV16 E6, and HPV16 E7 stains, cells were fixed in 2% paraformaldehyde and permeabilized with 0.1% Triton X-100. The same condition was also used for cells that were only stained with DAPI for nuclear size assessment. For RB1 stain, cells were fixed and permeabilized in cold 3:1 methanol/acetic acid mix. BrdU stain is described in *BrdU Labeling* (see below); 10% normal goat serum was used for blocking after permeabilization. Primary antibodies were diluted in 0.1% Triton X-100/PBS and incubated with the cells overnight at 4°C. Signals were detected by a corresponding Alexa Fluor 488–conjugated secondary antibody (ThermoFisher Scientific). DAPI was used to stain the nuclei.

### Western Blot Analysis

Cells were lysed in 1× SDS buffer [2% SDS, 50 mmol/L Tris (pH 6.8), 10% glycerol, 2.5 mmol/L sodium pyrophosphate, 1 mmol/L β-glycerophosphate, 1 mmol/L Na_3_VO_4_ (1 μg/mL), and SigmaFAST protease inhibitor], followed by brief sonication. The Western blot procedures were performed as described.^38^ The protein signal was detected by a horseradish peroxidase–conjugated secondary antibody and developed with ECL reagent (number 34095; ThermoFisher Scientific). Independent blots were used when lysates needed to be probed with multiple antibodies. The blots that had little possibility for signal interference from previous probing were stripped and used for loading controls. ImageJ version 2.0.0-rc-43/1.52n software (NIH, Bethesda, MD; *https://imagej.nih.gov/ij/download.html*, last accessed September 1, 2020) was used to determine the integrated density of the protein relative to tubulin.

### Scratch Assay

The scratch assay was performed and measured on the basis of the published protocol.^42^ A total of 10^6^ cells were seeded on a 35-mm culture dish in triplicate. On the following day, a p200 pipet tip was used to generate a straight scratch across the cell monolayer. The detached floating cells and debris were removed by rinsing with PBS, and the media were replenished. Three reference points close to each scratch were marked with a fine-tip permanent marker. Images of the scratch were taken at these points at time 0 and 8 hours later. The scratch areas were measured with QuPath software version 0.2.3.^43^

### Apoptosis (TUNEL) Stain and Hydrogen Peroxide–Induced Cell Death

A total of 10^5^ cells were seeded per glass coverslip and placed in 24-well plates in triplicate. The apoptosis signals were detected by terminal deoxynucleotidyl transferase-mediated dUTP nick-end labeling (TUNEL) stain using ApopTag Peroxidase *in Situ* Apoptosis Detection Kit (number S7100; MilliporeSigma), according to the manufacturer's instructions. This kit was also used on mouse tissue sections to detect apoptosis. The apoptosis stain was performed on tissues derived from six mice per analysis group. Hydrogen peroxide was used to induce cell death by genotoxic effects. The cells were cultured in 96-well plates (1000 cells/well) and treated with media containing 0, 100, and 200 μmol/L H_2_O_2_ for 1 hour the following day. The viability of the treated cells was quantified with a CellTiter-Glo Luminescent Cell Viability Assay kit (number G7570; Promega, Madison, WI) following the manufacturer's instructions.

### BrdU Labeling

A total of 10^5^ cells were plated per glass coverslip in triplicate and labeled with 3 μmol/L BrdU (number B5002; MilliporeSigma) for 30 minutes the following day. The cells were then fixed with cold 70% ethanol at −20°C for 1 hour, followed by incubation with 2 N HCl 0.3% Triton X-100 to denature the DNA at room temperature for 30 minutes. BrdU signals were detected by immunofluorescence staining (described above). For *in vivo* BrdU labeling, mice received an i.p. injection of BrdU (100 μL, 10 mg/mL) 24 hours before euthanasia. The heads were harvested and processed. The derived tissue sections were subjected to immunohistochemical staining (see below) with an anti-BrdU antibody. The staining was performed on tissues derived from six mice per analysis group.

### Tissue Processing and Staining

The harvested tissues were fixed in 10% formalin for 3 days at room temperature before placing in 10% EDTA for decalcification at 4°C for 3 weeks. These tissues were paraffin embedded and cut into sections (5 μm thick). The sections were transferred to glass sides and subjected to standard hematoxylin and eosin staining or special staining as indicated.

### IHC Data

All immunohistochemistry (IHC) procedures were conducted in the presence of appropriate positive and negative controls. Standard staining procedures were used following the manufacturer's instructions for ImmPRESS reagents (Vector Laboratories, Burlingame, CA). The antibody dilution and antigen retrieval conditions are detailed in Table 1. For IHC stains that used a single antibody, diaminobenzidine (number SK-4103; Vector Laboratories) was used as the horseradish peroxidase chromogenic substrate, and hematoxylin was used as the counterstain. For the FOXP3-CD4 double IHC stain, the FOXP3 IHC was performed first, and 5-Bromo-4-chloro-3-indolyl phosphate/nitro blue tetrazolium (number SK-5400; Vector Laboratories) was used as the alkaline phosphatase chromogenic substrate. This was followed by the CD4 IHC, with AMEC (number SK-4285; Vector Laboratories) as the horseradish peroxidase chromogenic substrate. Fast Green FCF was used as the counterstain. The staining was performed on tissues derived from six mice per analysis group.

**Table 1:** Antigen Retrieval and Antibody Dilution Conditions Used for IHC

Antibody	Dilution	Antigen retrieval
CD4	1:800; Single stain (DAB), double stain (AMEC)	10 mmol/L Tris and 1 mmol/L EDTA, pH 9.0; pressure cooker, 1 minute
CD8	1:3200	
FOXP3	Single stain (DAB): 1:200; double stain (BCIP/NBT): 1:1800.	
BrdU	1:1600	10 mmol/L Sodium citrate and 0.05% Tween 20, pH 6.0; pressure cooker, 2 minutes
ITGA2	1:200	
ARG1	1:1800	
NOS2	1:2000	
CD68	1:200	Proteinase K (40 mg/mL), 37°C for 20 minutes
F4/80	1:200	

ARG1, arginase 1; BCIP/NBT, 5-Bromo-4-chloro-3-indolyl phosphate/nitro blue tetrazolium; BrdU, bromodeoxyuridine; DAB, diaminobenzidine; FOXP3, forkhead box P3; IHC, immunohistochemistry; ITGA2, integrin subunit alpha 2; NOS2, nitric oxide synthase 2.

### HPV RNA *in Situ* Hybridization

HPV RNA *in situ* hybridization was performed using RNAScope 2.5 LS Probe - HPV16/18 (number 311128; Advanced Cell Diagnostics. Newark, CA), according to manufacturer's instructions. The assay was performed on Leica BondRx (Buffalo Grove, IL), and Fast Red was the chromogen counterstained by hematoxylin.

### Image Processing and Analysis

The acquired images were analyzed using QuPath software 0.2.3.^43^ For immunofluorescence stains, the percentage of positive cells among total cells (determined by DAPI staining) was calculated. Nuclear sizes were also determined. For IHC- and TUNEL-stained tissue sections, multiple nonoverlapping images were taken to cover the entire lesion in the section. QuPath was used to assess the signals within the lesion, which was defined as the E6/E7-expressing epithelium and the adjacent connective tissue stroma. The surrounding skeletal muscle areas were not included in the assessment. Positive cells were identified by intensities of mean nuclear staining or mean cytoplasm staining, depending on the subcellular localization of the staining target. The number of total cells was based on the mean nuclear signal of hematoxylin counterstain. The number of positive cells per mm^2^ was calculated by the software. Six mice were included in each analysis group.

### Statistical Analysis

Data were analyzed using an unpaired two-tailed *t*-test and were expressed as means ± SD; *P* ≤ 0.05 is considered significant.

## Results

### *In Vitro* Characterization of HPV16 E6/E7 Immortalized Mouse Oropharyngeal Keratinocyte Cell Lines

**Figure 1:**
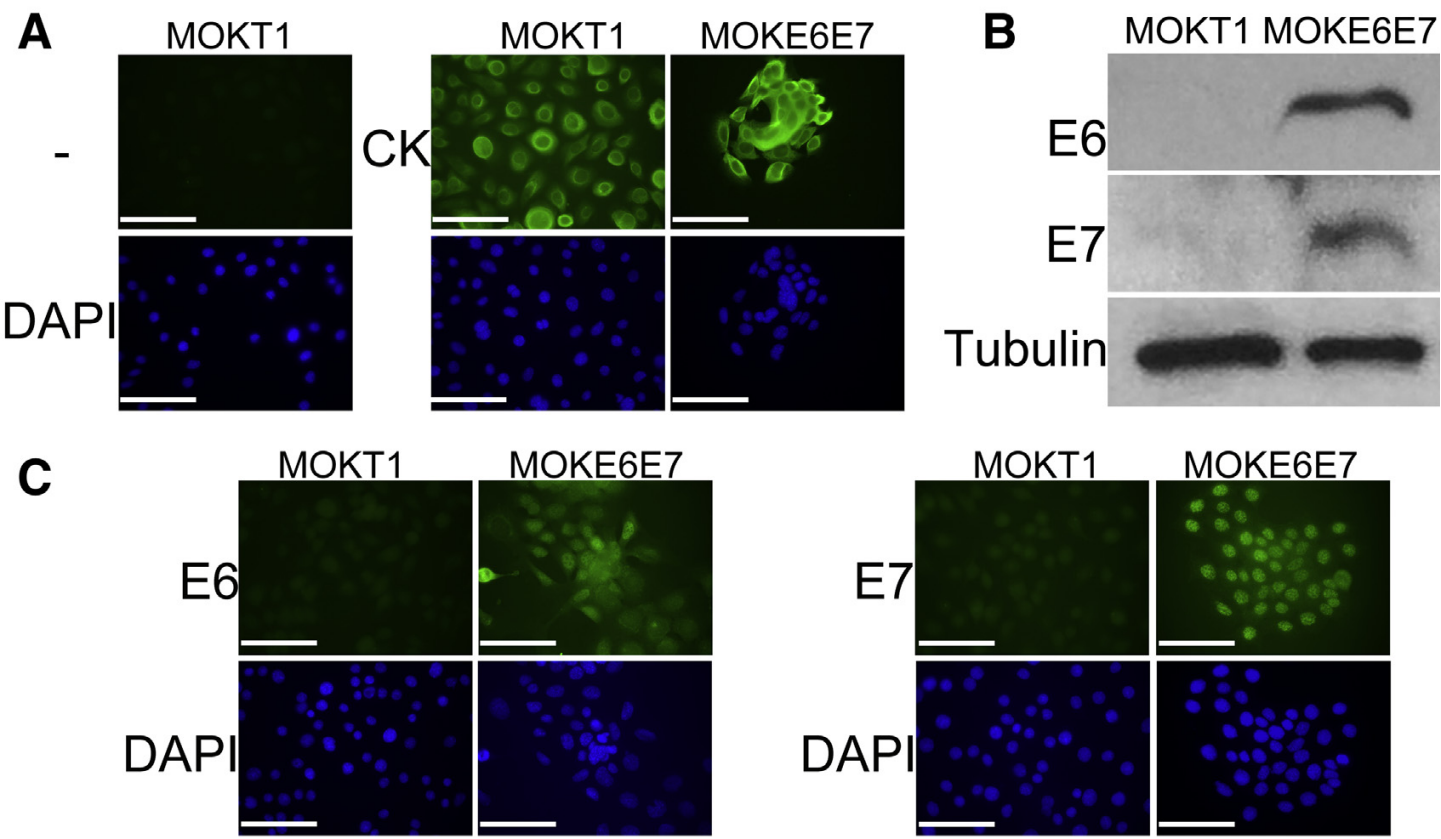
Establishment of C3H mouse oropharyngeal cell lines MOKT1 and MOKE6E7. **A:** Immunofluorescence staining of pan-cytokeratin (CK) confirms the keratinocyte identity. Negative control (–): secondary antibody only. **B:** Western blot analyses confirms the expression of HPV16 E6 and E7. Tubulin was a loading control. **C:** Immunofluorescence staining confirms the expression of HPV16 E6 and E7. DAPI was used to stain nuclei. The data are representative of three independent experiments. Scale bar = 100 μm (**A** and **C**). Original magnification, ×400 (**A** and **C**).

HPV16 is the predominant genotype found in HPV-positive OPSCC. HPV16 E6/E7 were used to immortalize the mouse tongue keratinocytes to establish the MOKE6E7 cell line. This was used as a premalignant cell line because a concurrent second genetic alteration was required for the cells to form permanent tumors *in vivo*. A nonneoplastic control cell line MOKT1, immortalized by hTERT, was also established. As indicated by immunofluorescence staining, all of the cells in the two cell lines expressed cytokeratin, which confirmed the keratinocyte identity and ruled out the presence of nonepithelial components (Figure 1A). E6 and E7 expression was confirmed by Western blot analysis (Figure 1B) and immunofluorescence (Figure 1C). In MOKE6E7, the subcellular localization of E6 and E7 was either nuclear, cytoplasmic, or both. Figure 1C shows predominantly nuclear localization of E6 and E7 in the MOKE6E7 cells.

**Figure 2:**
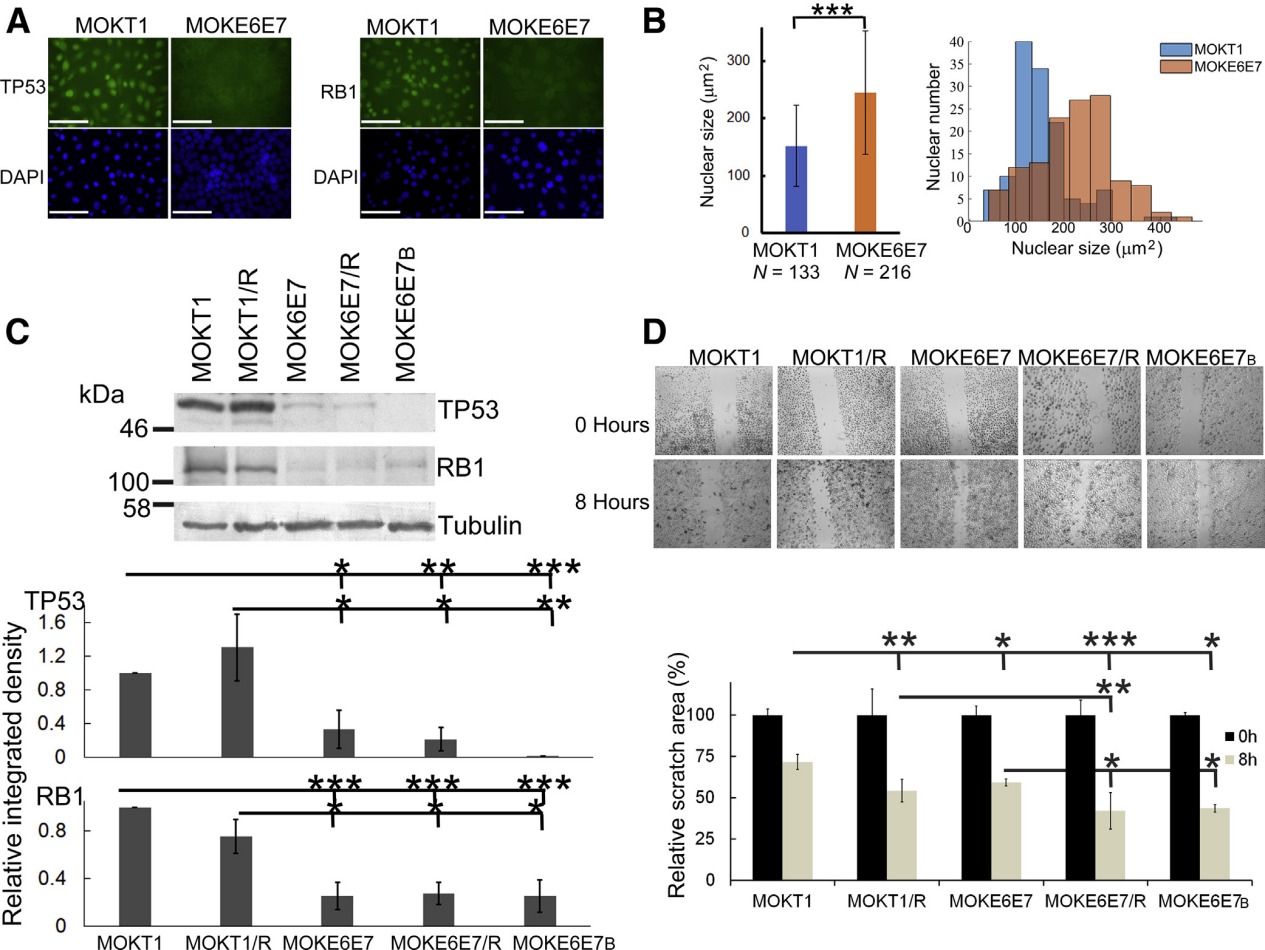
Expression of HPV16 E6/E7 changes the cellular status of keratinocytes. **A:** Immunofluorescence staining of TP53 and retinoblastoma in MOKT1 and MOKE6E7 cells. DAPI was used to stain nuclei. **B:** MOKT1 versus MOKE6E7. **Left panel:** Nuclear sizes. **Right panel:** Distribution of the nuclear sizes. **C:** Western blot analyses of TP53 and RB1 in MOKT1- and MOKE6E7-derived cell lines. Tubulin is the loading control. The quantitative results are from three independent lysates in three independent experiments. The protein levels of TP53 and RB1 are expressed as relative integrated density (normalized to the levels of tubulin). **D:** Scratch assay of the MOKT1- and MOKE6E7-derived cell lines. **Top panels:** Scratches of cell monolayers at time 0 and 8 hours later. Scratch assay was performed in triplicate. **Bottom panel:** The scratch areas on the entire plate were quantified, and the changes between the time points were calculated and plotted. The data are representative of three independent experiments. ∗*P* ≤ 0.05, ∗∗*P* ≤ 0.01, and ∗∗∗*P* ≤ 0.001. Scale bar = 100 μm (**A**). Original magnifications, ×400 (**A**); ×40 (**D**). *N*, number of nuclei counted.

**Supplemental Figure S1:**
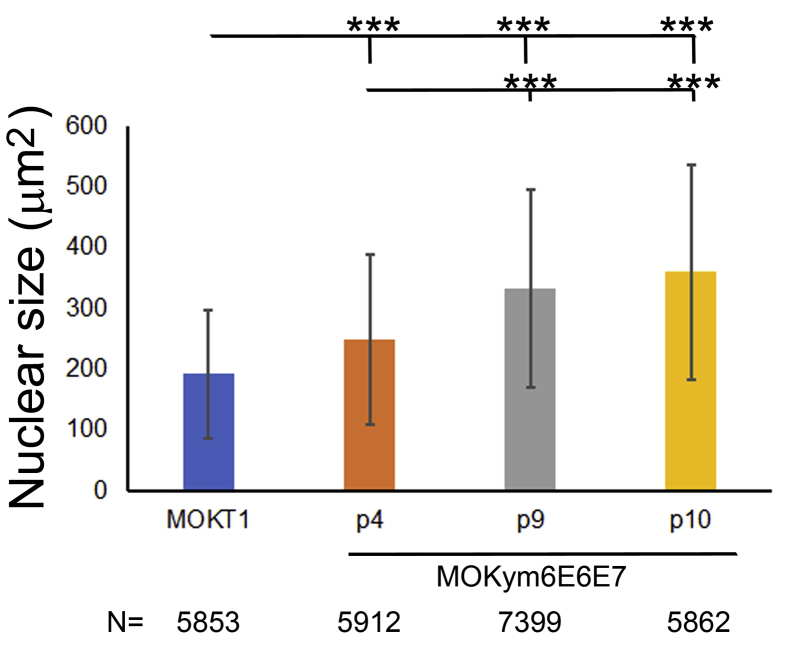
HPV16 E6E7 immortalized keratinocytes exhibit nuclear size changes early in the passage. MOKym6E6E7 cells are mouse oropharyngeal keratinocytes immortalized by HPV16 E6E7, established independently from MOKE6E7 cells. The results are compiled from three independent experiments. Data represent means ± SD. ∗∗∗*P* ≤ 0.001. N, number of nuclei counted; p, passage number.

The status of TP53 and RB1 was examined to validate the inhibitory functions of E6 and E7 in the newly established cell line MOKE6E7. Immunofluorescence results showed that although strong nuclear signals of TP53 and RB1 were present in control MOKT1 cells, they were significantly down-regulated in MOKE6E7 cells (Figure 2A). High-risk HPV is known to cause genomic instability,^44^ which can lead to the accumulation of genetic material and subsequent increase in nuclear sizes.^45^ In contrast to MOKT1 cells, MOKE6E7 cells had larger nuclei and exhibited a wider distribution of nuclear sizes (Figure 2B). HPV16-immortalized human oral keratinocytes exhibit detectable trisomy events in passage 10 and polyploidy/aneuploidy by passage 61.^46^ Here, nuclear size changes of HPV16 E6/E7-expressing keratinocytes were detectable in even earlier passages (Supplemental Figure S1).

This study was intended to evaluate the stable, long-term epigenetic effects of butyrate on oropharyngeal keratinocytes immortalized by HPV oncoproteins. To maximize the chemical effects on chromatin within a short time, MOKE6E7 cells were treated with 20 mmol/L butyrate for 48 hours to produce MOKE6E7_B_ cells.^47^^,^^48^ Epigenetic modifications can be short-term or long-term. Because long-term epigenetic modifications are expected to be stable across generations, even in the absence of the original inducer, a range of the successive MOKE6E7_B_ passages was used in the study after butyrate was withdrawn from the culture media. Two additional cell lines, MOKE6E7/R and MOKT1/R, were established. MOKE6E7/R was established as a growth control line by expressing HRasV12 in MOKE6E7. HRasV12 is a constitutively active form of HRas and was used as a surrogate oncogene. The control line, MOKT1/R, was established by expressing HRasV12 in the MOKT1 cells. Butyrate is a potent HDAC inhibitor and a common metabolite produced by anaerobic bacteria, including those that inhabit the tonsillar crypts. Expression of HRasV12 or butyrate treatment did not revert the abilities of HPV16 E6 and E7 to down-regulate TP53 and RB1 (Figure 2C).

Cell migration allows the tumor to disseminate and is linked to the invasive potential of tumor cells. Scratch assay, involving testing the ability of cells to close the gaps generated by tip scratching within 8 hours, was used to evaluate the ability of the newly established cell lines to migrate (Figure 2D). Both E6/E7 and HRasV12 expression levels increased cell migration, and additive effects between these oncogenes were noted. Butyrate treatment also mildly increased cell migration (MOKE6E7_B_ versus MOKE6E7).

### Butyrate Exposure Changes the Growth Pattern and Increases the Survival Time of HPV16 E6/E7-Immortalized Keratinocytes *in Vivo*

The newly established oropharyngeal cell lines were injected into the masseter muscle of syngeneic immunocompetent C3H mice, and the resulting lesions were harvested at various time points for analysis.

**Figure 3:**
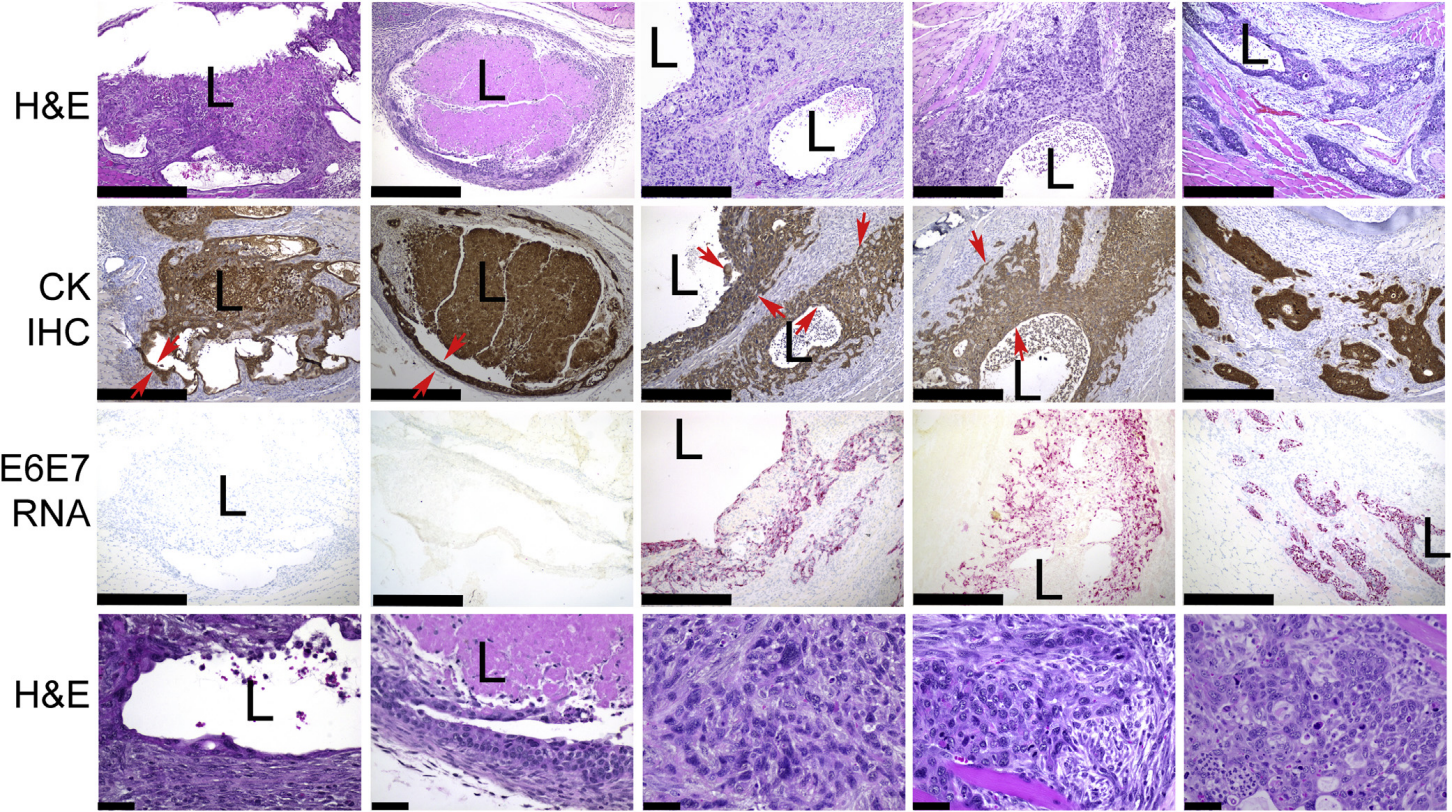
Histological analyses of day 4 lesions formed by the newly established cell lines in C3H mice. **Row 1:** Hematoxylin and eosin (H&E) stain shows that all the injected cell lines form lesions with cystic components. MOKT1 and MOKT1/R form cysts filled with necrotic debris. MOKE6E7 and MOKE6E7/R form large cysts, with lining epithelium proliferated around the lumens. MOKE6E7_B_ lesions are composed of scattered epithelial islands with microcystic changes. **Row 2:** Pan-cytokeratin (CK) immunohistochemistry (IHC) confirms that the lesions are composed of epithelium. **Red arrows** mark the cystic linings. **Row 3:** RNAScope confirms the expression of HPV16 E6/E7 RNA in lesions formed by MOKE6E7, MOKE6E7/R, and MOKE6E7_B_ cells. **Row 4:** H&E stain shows normal-looking squamous keratinocytes in hTERT-expressing epithelium (MOKT1 and MOT1/R) versus basaloid keratinocytes with marked cytologic atypia in E6/E7-expressing epithelium (MOKE6E7, MOKE6E7/R, and MOKE6E7_B_). Images are representative of the studied mice in the group. *N* = 6 per group. Scale bars: 500 μm (**rows 1 to 3**); 50 μm (**row 4**). Original magnifications, ×100 (**rows 1 to 3**); ×400 (**row 4**). L, cystic lumen.

All five cell lines formed cystic structures 4 days after injection (Figure 3). Cytokeratin IHC demonstrated that the cysts were lined by stratified squamous epithelium (Figure 3). RNAScope validated the expression of HPV16 E6/E7 RNA (Figure 3). The presence of HRasV12 did not result in any neoplastic features for MOKT1/R, and both hTERT-expressing cell lines formed cysts filled with necrotic debris in the lumens, which were lined by thin epithelium with normal cytology (Figure 3).

**Figure 4:**
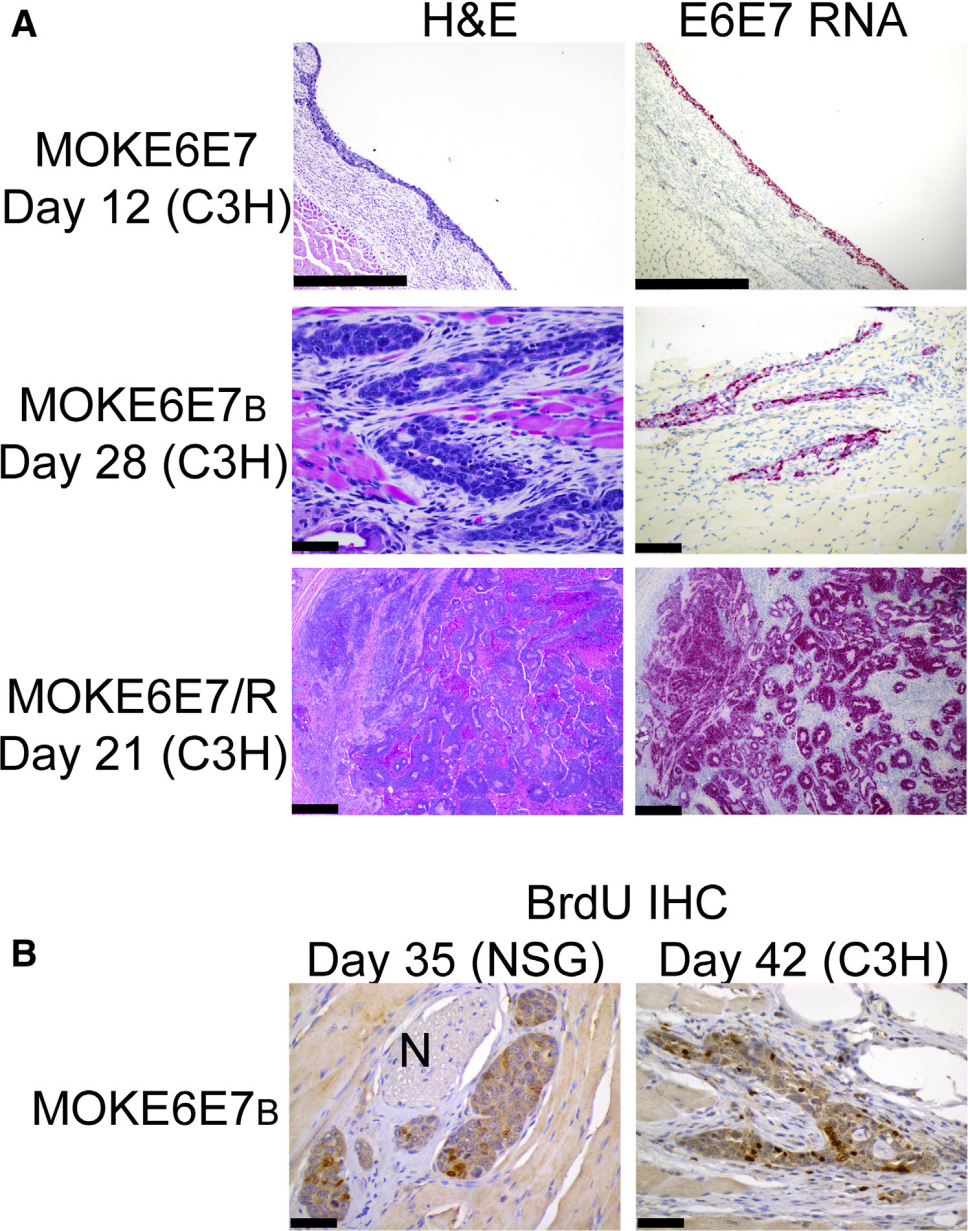
HPV16 E6/E7-expressing keratinocytes gain survival advantages after butyrate treatment, whereas formation of a long-term tumor requires the presence of a second oncogenic event. **A:** Hematoxylin and eosin (H&E) stain and RNAScope of HPV16 E6/E7 performed on lesions formed by MOKE6E7, MOKE6E7_B_, and MOKE6E7/R cells in C3H mice. **Top panels:** Day 12 MOKE6E7-derived lesions, when the lesion was last detected. **Middle panels:** Day 28 MOKE6E7_B_-derived lesion. **Bottom panels:** Day 21 MOKE6E7/R-derived lesion, with tumor formation. **B:** Positive bromodeoxyuridine (BrdU) signals in MOKE6E7_B_-derived epithelium in nonobese diabetic–severe combined immunodeficiency IL-2Rγnull (NSG; day 35) and C3H (day 42) mice. Images are representative of the studied mice in the group. *N* = 6 per group. Scale bars: 500 μm (**A**, **top** and **bottom panels**); 50 μm (**A**, **middle panels**, and **B**). Original magnifications, ×100 (**A**, **top panels**); ×400 (**A**, **middle panels**, and **B**); ×40 (**A**, **bottom panels**). IHC, immunohistochemistry.

In contrast, all three HPV16 E6/E7 cell lines differentiated into nonkeratinized, basaloid epithelium with marked mitotic activity (Figure 3), reminiscent of the tumor epithelium in HPV-positive OPSCC. The microscopic sections derived from the day 4 specimens show that MOKE6E7 and MOKE6E7/R formed large cysts, with lining epithelium proliferated around the lumens (Figure 3). MOKE6E7_B_, however, exhibited a different growth pattern. Instead of a macrocytic configuration, MOKE6E7_B_ lesions were composed of scattered epithelial islands with microcystic changes, without a clear growth center(s). In later samples, both MOKE6E7 and MOKE6E7_B_ lesions reduced in size, although at different rates and appearance. On day 12, the MOKE6E7 keratinocytes became flat and small, and the lesion contracted to a cyst with thin lining and minimal numbers of rete ridges (Figure 4A). Within 2 weeks, the MOKE6E7 cyst completely regressed without any remnant. In contrast, the MOKE6E7_B_ keratinocytes did not significantly change their sizes. Although MOKE6E7_B_ also developed prominent cystic spaces on day 12, small epithelial nests/islands often were the only form that persisted before regressing. Metabolically active MOKE6E7_B_ epithelial islands, as evident by the positive BrdU labeling, were last detectable 5 to 6 weeks after injection (Figure 4B). The survival time of MOKE6E7_B_ cells in the immunocompromised nonobese diabetic–severe combined immunodeficiency IL-2Rγnull mice was not significantly different from that in the immunocompetent C3H mice.

Of the cell lines injected into the mice, only MOKE6E7/R formed persistent solid tumor masses (Figure 4A). *HRas* mutations are not typically associated with HPV-positive OPSCC but are commonly present in oral SCC, with or without the presence of HPV.^49^^,^^50^ *HRasV12* was used as a surrogate oncogene to demonstrate that HPV16 E6/E7 expression in keratinocytes induced sufficient cellular changes that led to persistent tumor masses in the presence of a second oncogenic hit.

### HPV16 E6/E7-Immortalized Keratinocytes with Butyrate Exposure Show Enhanced Resistance to Cell Death, Decreased Proliferation, and Improved Differentiation

**Figure 5:**
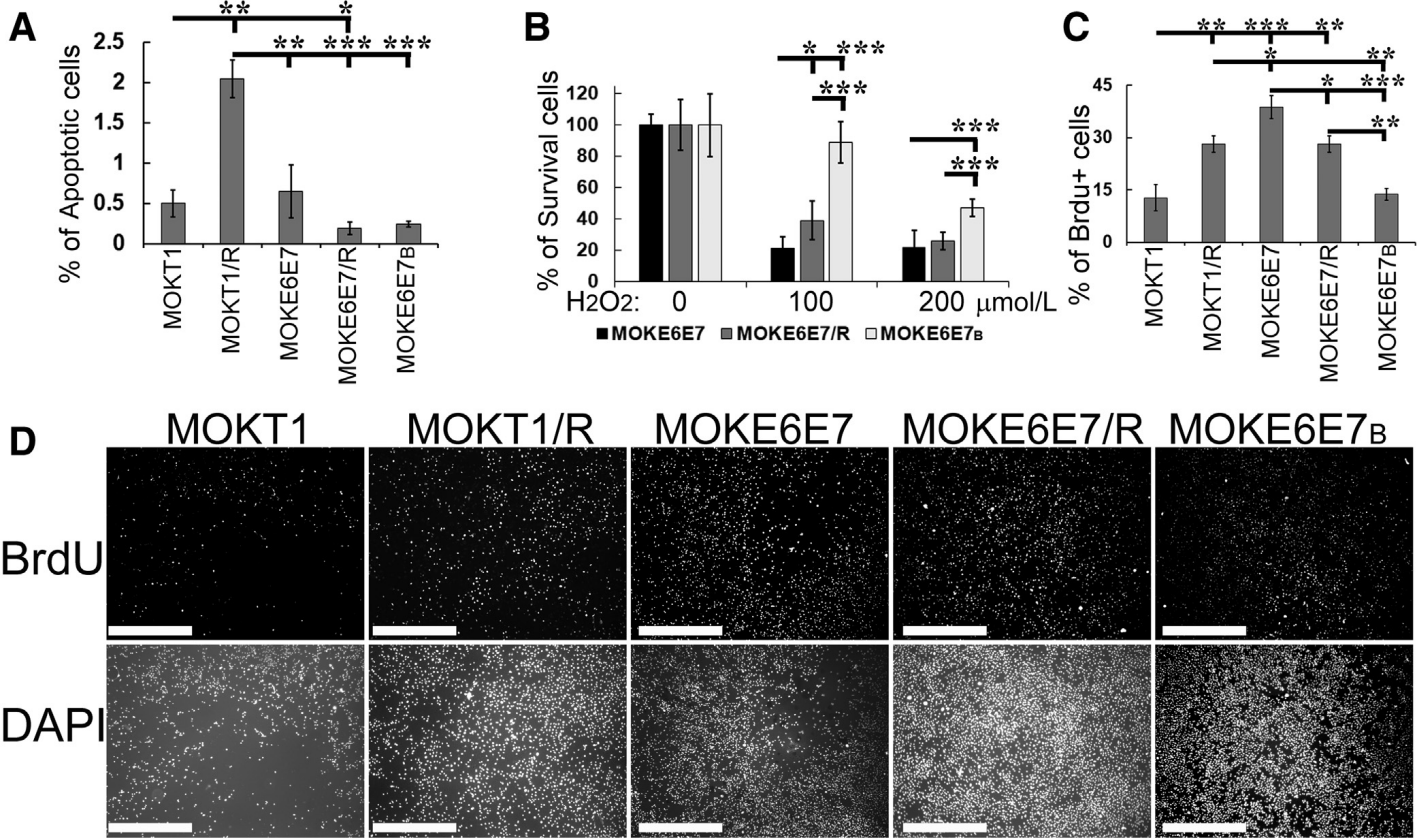
Effects of butyrate treatment and HRasV12 expression on cell death and proliferation. **A:** Terminal deoxynucleotidyl transferase-mediated dUTP nick-end labeling assay of the cultured MOKT1- and MOKE6E7-derived cell lines. Micrographs are shown in Supplemental Figure S2. The apoptotic rate is calculated by dividing the number of positive nuclei by the number of total nuclei (determined by DAPI). **B:** Luminescent cell viability assay after 1 hour of hydrogen peroxide treatment. The survival rate is calculated by dividing the cell numbers of the hydrogen peroxide–treated group by those of the untreated group. **C:** Quantification of bromodeoxyuridine (BrdU) immunofluorescence stain (see **D**). Cell proliferation rates of the newly established cell lines were assessed by BrdU incorporation. The proliferation rate is calculated by dividing the number of BrdU^+^ nuclei by total nuclei. **D:** BrdU immunofluorescence stain (quantified in **C**). The data are representative of three independent experiments. ∗*P* ≤ 0.05, ∗∗*P* ≤ 0.01, and ∗∗∗*P* ≤ 0.001. Scale bar = 1 mm (**D**). Original magnification, ×40 (**D**).

**Supplemental Figure S2:**
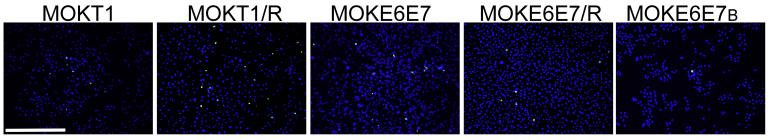
Terminal deoxynucleotidyl transferase-mediated dUTP nick-end labeling stain. Immunofluorescence micrographs of Figure 5A. Apoptosis: green. Nuclei: blue (DAPI). Butyrate treatment and HRasV12 expression have little effect on the baseline of apoptosis in HPV16 E6E7-expressing keratinocytes, although HRasV12 expression significantly increases apoptosis in keratinocytes expressing hTERT. Scale bar = 500 μm. Original magnification, ×100.

Similar to hTERT, HPV16 E6/E7 also inhibit apoptosis.^51^^,^^52^ Consequently, both MOKT1 and MOKE6E7 cell lines exhibited low levels of apoptosis *in vitro* (Figure 5A and Supplemental Figure S2). HRasV12 expression in hTERT cells significantly increased apoptosis (MOKT1/R versus MOKT1). In contrast, HRasV12 expression (MOKE6E7/R) and butyrate treatment (MOKE6E7_B_) had no significant effects on the apoptotic rates (versus MOKE6E7). The indifference of apoptotic rates among the three MOKE6E7-derived cell lines likely was attributable to their low baseline levels of apoptosis. Recent studies have shown the importance of chronic inflammation and oxidative stress in the pathogenesis of HPV infection and carcinogenesis.^53^^,^^54^ HPV-related cancers often have excessive inflammation, and the cancer cells commonly shift to a pro-oxidative condition. Therefore, these three cell lines were challenged with hydrogen peroxide–induced genotoxicity to examine their abilities to survive an oxidant microenvironment. The percentage of the cells surviving the hydrogen peroxide exposure represented their ability to survive oxidative stress. The results showed that MOKE6E7_B_ and MOKE6E7/R had better short-term survival rates than their parental cell, MOKE6E7 (Figure 5B). Whether the short-term survival advantage extends to long-term survival remains to be determined.

Among the five cell lines, MOKE6E7 had the highest BrdU incorporation rate, suggesting the highest proliferation potential (Figure 5, C and D). Interestingly, both HRasV12 expression (MOKE6E7/R) and previous exposure to butyrate (MOKE6E7_B_) reduced the cell proliferation rates (versus MOKE6E7). In contrast to that in E6/E7-expressing cells, HRasV12 expression in the hTERT-expressing cell line increased the proliferation rate (MOKT1/R versus MOKT1) (Figure 5, C and D).

**Figure 6:**
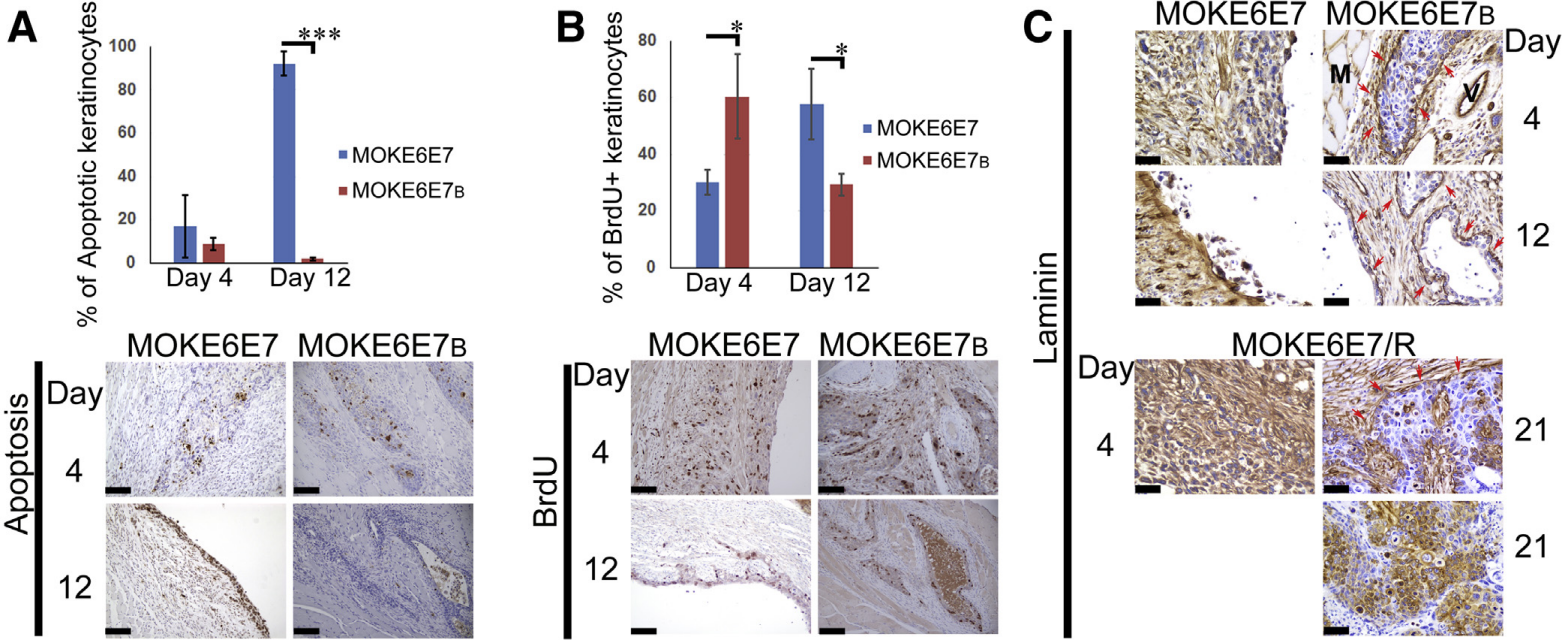
Butyrate treatment promotes survival and improves cell differentiation of HPV16 E6/E7-expressing epithelium. **A:** Quantification and micrographs of terminal deoxynucleotidyl transferase-mediated dUTP nick-end labeling stain in MOKE6E7- and MOKE6E7_B_-derived lesions. The apoptotic rate is calculated by dividing the number of positive keratinocyte nuclei by the number of total keratinocyte nuclei within the lesion. **B:** Bromodeoxyuridine (BrdU) immunohistochemistry (IHC) labels proliferating cells *in vivo*. The proliferation rate is calculated by dividing the number of positive keratinocyte nuclei by the number of total keratinocyte nuclei within the lesion. **C:** Laminin IHC stain. **Red arrows** mark the basement membrane. Images are representative of the studied mice in the group. Data represent means ± SD (**A** and **B**). *N* = 6. ∗*P* ≤ 0.05, ∗∗∗*P* ≤ 0.001. Scale bars: 100 μm (**A** and **B**); 50 μm (**C**). Original magnifications, ×200 (**A** and **B**); ×400 (**C**). M, muscle; V, vessel.

The effects of butyrate on HPV16 E6/E7-expressing keratinocytes were further characterized *in vivo*. TUNEL stain for apoptosis and BrdU IHC for cell proliferation were performed on the tissue sections derived from syngeneic mice receiving injections of MOKE6E7 or MOKE6E7_B_ cells. The results showed that although both lesions exhibited low apoptotic rates on day 4, the rate escalated to 92% for the MOKE6E7 cells, whereas it remained low for the MOKE6E7_B_ cells on day 12 (Figure 6A). Both cell lines exhibited considerable proliferation abilities within this time period (Figure 6B), and MOKE6E7 quickly regressed afterward. Evidently, MOKE6E7's proliferation rate was insufficient to compensate for its exceptionally high apoptotic rate. At the same time, MOKE6E7_B_ was able to persist for a much longer time because of a lower apoptotic rate.

Although all three MOKE6E7-derived cell lines produced epithelium with high-grade, basaloid morphology in mice, their degrees of differentiation were different. Although the MOKE6E7-derived epithelium never developed an organized basement membrane, the MOKE6E7_B_-derived epithelium was always bounded by a continuous basement membrane, as demonstrated by the laminin IHC stain (Figure 6C). The epithelium also had a clear cell-cell boundary. For MOKE6E7/R-derived epithelium, an organized basement membrane was not present on day 4, but could be found around some, but not all, tumor islands at later stages (Figure 6C).

### Presence of Immune Regulatory Cells in Lesions Formed by HPV16 E6/E7-Immortalized Keratinocytes

**Figure 7:**
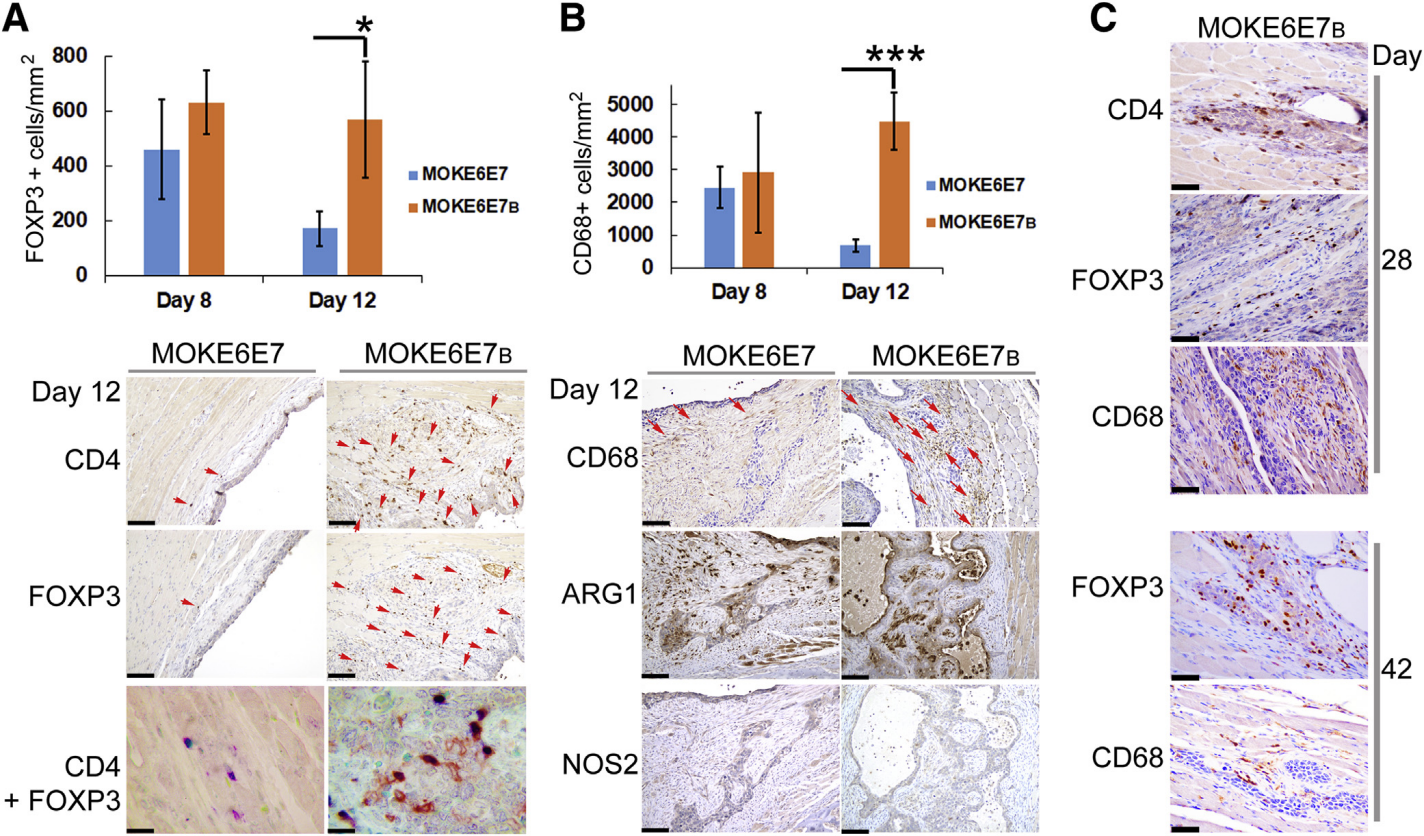
Presence of immune regulatory cells in lesions formed by HPV16 E6/E7-expressing keratinocytes. **A: Top panel:** FOXP3^+^ cell density on day 8 and 12 lesions formed by MOKE6E7 and MOKE6E_B_ cells. **Bottom panels:** CD4 and FOXP3 immunohistochemistry (IHC) analyses on the day 12 lesions. CD4 and FOXP3 IHC analyses: **Red arrows** mark positive brown signals. FOXP3/CD4 double IHC: Nuclear blue color represents FOXP3 signals, and membranous red color represents CD4 signals. Almost all FOXP3^+^ cells detected in both lesions were also CD4^+^, which represented regulatory T cells, but not vice versa. **B: Top panel:** CD68^+^ cell density on day 8 and 12 lesions. **Bottom panels:** CD68, arginase 1 (ARG1), and nitric oxide synthase (NOS) 2 IHC analyses on the day 12 lesions. **Red arrows** mark the CD68 signals. Macrophages present in the lesions are polarized to M2. **C:** CD4, FOXP3, and CD68 IHC analyses on day 28 and 42 lesions of MOKE6E7_B_. Images are representative of the studied mice in the group. Data represent means ± SD (**A** and **B**). *N* = 6. ∗*P* ≤ 0.05, ∗∗∗*P* ≤ 0.001. Scale bars: 100 μm (**A**, **bottom panels**, CD4 and FOXP3 IHC analyses, and **B**, **bottom panels**); 20 μm (**A**, **bottom panels**, FOXP3/CD4 double IHC); 50 μm (**C**). Original magnifications, ×200 (**A**, **bottom panels**, CD4 and FOXP3 IHC analyses, and **B**, **bottom panels**); ×1000 (**A**, **bottom panels**, FOXP3/CD4 double IHC); ×400 (**C**). FOXP3, forkhead box P3.

**Supplemental Figure S3:**
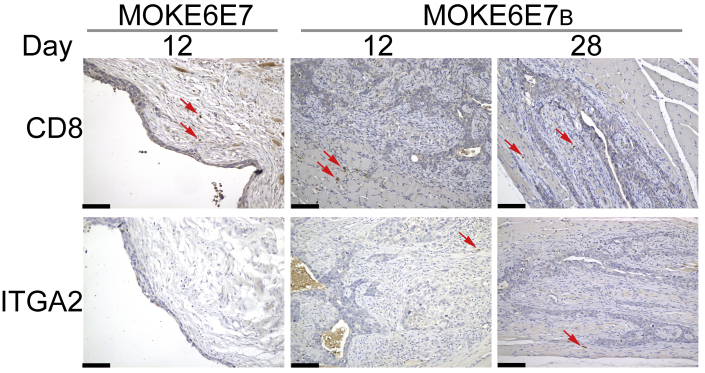
Absence of CD8 or Integrin Subunit Alpha 2 signals in the MOKE6E7 and MOKE6E7_B_ lesions. CD8 and Integrin Subunit Alpha 2 immunohistochemistry analyses. **Red arrows** mark positive brown signals. Scale bar = 100 μm. Original magnification, ×200.

Butyrate, like many HDAC inhibitors, has immune modulation capabilities.^55^ However, it was unclear if a one-time, *in vitro* treatment of butyrate would affect the keratinocytes' ability to recruit immune cells. IHC analyses showed that although there was no significant presence of CD8 or ITGA2 signals (Supplemental Figure S3), considerable signals of immune marker antibodies CD4, FOXP3, CD68, and F4/80 became detectable 8 days after the initial injection (Figure 7). Almost all FOXP3^+^ cells detected in both lesions were also CD4^+^, which represented regulatory T cells, but not vice versa (CD4^+^FOXP3 double IHC) (Figure 7A). Both MOKE6E7- and MOKE6E7_B_-derived lesions recruited substantial numbers of CD4^+^ and FOXP3^+^ cells on day 8. On day 12, the density of regulatory T cells, represented by the FOXP3^+^ signals, declined in MOKE6E7-derived lesions, but remained unchanged in MOKE6E7_B_-derived lesions. Day 12 was also the time point for MOKE6E7 keratinocytes to develop near-universal apoptosis (Figure 6A).

Unexpectedly, both lesions showed a significant presence of eosinophils, which interfered with the interpretation of F4/80 IHC results because this marker is shared by both macrophages and eosinophils. Hence, an alternative macrophage marker, CD68, was used for the IHC studies on macrophages. On day 8, high and comparable densities of macrophages were present in both lesions (Figure 7B). But on day 12, although the macrophage density in MOKE6E7_B_-derived lesion remained substantial, the number decreased significantly for MOKE6E7-derived lesions, similar to the findings for regulatory T cells. The polarity of these infiltrated macrophages was further examined. Although the ARG1 signals were not exclusively from macrophages, all specimens exhibited strong ARG1 and undetectable nitric oxide synthase 2 signals (Figure 7B). ARG1 and nitric oxide synthase 2 are common markers for M1/M2 detection, although their sole presence is not indicative of the polarization. Nevertheless, in the current case, the combination of strong ARG1 and a near-total absence of nitric oxide synthase 2 signals indicated that most of the macrophages in both lesions were polarized to M2. The MOKE6E7_B_-derived epithelium persistently recruited immune regulatory cells until the lesions regressed (Figure 7C).

## Discussion

Long-term survival of HPV-infected cells is crucial for the development of HPV-associated cancer. The persistent presence of the infected cells not only is necessary to maintain the oncogenesis but also allows accumulations of critical genetic alterations required for malignancy. HPV has evolved multiple countermeasures to evade host immunity,^56^ and the outcome of high-risk HPV infection is largely determined by the ability of the infected cells to persist. Like most cancers, HPV-associated oncogenesis is a slow, multistep process. Although keratinocytes can be immortalized and gain a high-grade morphology when expressing HPV E6/E7 oncoproteins, HPV infection alone is insufficient for cancer development.^57^ Second hits of genetic and/or epigenetic alterations are needed to provide additional collaborative changes to drive cancer development.

Given that host immunity can clear HPV infection in most cases, the question is why/how the infected keratinocytes persist in some patients but not others. The unusually high incidence of HPV-associated cancer in the oropharynx suggests that local factors may be responsible. The human oropharynx contains palatine and accessory tonsils, which form deep crypts, providing a low-oxygen habitat for anaerobic bacteria^24^^,^^25^ that produce a variety of byproducts, including butyrate.^26^ Because butyrate is a potent epigenetic modulator, this study investigated its potential role in the pathogenesis of HPV-positive OPSCC.

This study showed that a one-time *in vitro* exposure to butyrate was able to alter the characteristics of HPV oncoprotein-transformed keratinocytes *in vitro* and *in vivo*. Although thier stability is unclear, the effects appeared to last for several cell passages. These changes include easier adaptation to regular RPMI 1640 media, increased cell mobility, improved ability to resist environmental stress, slower growth, prolonged survival time, and altered growth architecture (Figure 3, Figure 4). Although influence from calcium and FBS concentration used in the media cannot be completely ruled out, the data suggest that butyrate treatment has an independent effect on the survival and differentiation of HPV immortalized keratinocytes.

Although MOKE6E7_B_ cells exhibited survival advantages over their parental cell line, MOKE6E7, they remained a transient lesion and disappeared around 5 to 6 weeks after injection. HPV16 E6/E7 immortalized cell lines could be considered premalignant, as they were able to form permanent tumors in the presence of a second oncogenic hit (Figure 4A).

OPSCC is known to develop cervical nodal metastases in the absence of morphologic features of conventional invasion, and *in situ* diagnosis is not used at this anatomic site for practical purposes. The lesions are often small and clinically occult, and patients frequently present with lymph node metastases compared with HPV-negative tumors that commonly present with pain.^58^ It is generally accepted that the lack of a continuous basement membrane of the reticular epithelium provides a weak barrier, allowing the HPV-infected cells to develop an invasive tumor front by budding off the basal cell layer with lesser restraint. In this study, MOKE6E7 cells showed no organized basement membrane *in vivo*, but they still formed epithelial lesions confluent with the keratinocytes around the main lumens, likened to the precursor lesions in the conventional SCC. In contrast, MOKE6E7_B_ cells produced scattered epithelial islands with cystic changes *in vivo*, which showed no clear origin for the growth. This observation suggests that the leaky basement membrane in the palatine tonsils is unlikely to be the main cause of rare encounters with OPSCC precursor lesions. Other factors, such as influence from the local environment, may have a more important role than previously identified.

Because epigenetic modulation plays a significant role in gene regulation and cell activities, microbiota-derived short-chain fatty acids, such as butyrate, have emerged as important epigenetic modulators for local cellular homeostasis. In particular, butyrate inhibits HDAC and consequently leads to increased levels of acetylation in chromatin histones, which lead to DNA unwinding and transcriptional activation. Butyrate also can cause DNA hypermethylation and demethylation by regulating DNA methyltransferase 1.^47^^,^^59^ Thus, cells exposed to butyrate have a broad range of changes in gene expression profiles, often leading to different cell activities or properties. Among the modifications that butyrate can induce on the chromatin, histone modifications are readily reversible, whereas DNA methylation is notable for its heritability and persistency even after removal of the inducer.^60^ Although other possibilities remain, DNA methylation is the most likely event responsible for the lasting phenotype of MOKE6E7_B_. Genome-wide approaches, such as bisulfite-treated whole genome sequencing, will be helpful to identify the epigenetic targets and mechanisms involved.

Butyrate has received increasing attention because of its potential beneficial impact on gut epithelia and has been proposed as a potential treatment for a wide variety of conditions, from chronic inflammatory diseases to cancer.^61^ In this study, MOKE6E7_B_ showed enhanced resistance to environmental stress, reduced proliferation, and improved differentiation, suggesting that the outcome of butyrate exposure is not always beneficial to the host. Interestingly, the expression of *HRasV12*, a constitutively active oncogene, also improved cellular differentiation and slowed down the proliferation of HPV16 E6/E7-expressing but not hTERT-expressing keratinocytes. Cellular pattern recognition receptors can detect HPV infection and activate the innate immune system, which often leads to an antiviral state and induces devastating events, such as apoptosis, seen in Figure 4.^62^ Although the mechanism remains unknown, butyrate treatment may have helped MOKE6E7_B_ to avoid massive apoptosis by modulating pattern recognition receptor responses. Thus, instead of promoting unrestricted activities induced by HPV E6/E7, genetic or epigenetic events that selectively fine-tune the balance between oncogenesis and survival may provide a better growth advantage for the infected cells in a hostile host environment.

HPV exhibits tropism for squamous epithelium and can infect oropharyngeal and oral mucosae. Unlike oropharyngeal SCC, only 1% of oral SCC harbors detectable high-risk HPV E6/E7 mRNA,^22^ despite the fact that squamous papilloma, an HPV lesion, is the most common benign neoplasm in the oral cavity.^63^^,^^64^ Obviously, cell tropism alone cannot explain the susceptibility of oropharyngeal keratinocytes to HPV-associated oncogenesis. In this location, the tonsils form deep and narrow crypts, providing a low-oxygen habitat, allowing the commensal anaerobic bacteria to thrive and their metabolites to accumulate. The anatomic proximity to lymphoid tissue and constant presence of lymphoid cells in the epithelium could also be important local factors. Normally, the lymphoid cells become activated after sensing viral infection, followed by killing and eliminating the infected cells. However, in the presence of butyrate, an HDAC inhibitor, the infected cells may have a better chance to survive as the environment could be modulated toward immune tolerant. Although more studies will be needed, the oropharyngeal commensal bacteria may play a role in the site preference of head and neck SCC.

During high-risk HPV infection, cellular expression of E6/E7 helps to recruit immune regulatory cells to the vicinity, which results in an immune-suppressive, protumoral microenvironment.^56^ In this study, MOKE6E7_B_, but not MOKE6E7, lesions were able to persistently maintain a close association with immune regulatory cells until regression (Figure 7). Because the densities of the immune regulatory cells were similar in both lesions at peak time on day 8, the differences in the ability to continue recruiting immune regulatory cells were unlikely to be related to the butyrate treatment. Instead, it is likely because MOKE6E7 developed massive cell death, which interferes with all cellular functions.

CD8^+^ T and natural killer cells did not appear to play significant roles in the dissolution of MOKE6E7 and MOKE6E7_B_ lesions, as both exhibited minimal CD8 and ITGA2 signals. In addition, an immunocompromised environment that lacks most of the immune cells did not appear to prolong the survival time of MOKE6E7_B_ (Figure 4). CD8^+^ T and natural killer cells are known for playing integral roles in the host defense system against HPV. Their absence may be related to the transient nature of the lesions developed in this study.

Because HDAC inhibition is known to affect tumor immunity, our inability to detect the effect likely was attributable to the study design because HDAC inhibition is reversible in the absence of the inducer.^60^ In the oropharynx, butyrate exposure is not limited to keratinocytes and is a chronic process. The crypts are lined by spongiotic epithelium that lacks an intact basement membrane, which presumably allows butyrate to permeate and affect all cells in the vicinity. As a result, not only the basal keratinocytes, where HPV-infected cells reside, but also the subjacent lymphoid tissue and stromal fibroblasts are exposed to butyrate.

This study explored the effects of butyrate exposure on cell survival and behavior using keratinocytes expressing steady-state levels of HPV oncogenes. Similar studies using the viral native promoter will be complementary and will add values to the current investigation by incorporating the environmental effects on the viral transcription to assess the overall epigenetic effects. The findings that butyrate exposure prolonged the survival of keratinocytes immortalized by HPV oncoproteins suggest that the commensal bacteria may have a potential role in the long-term persistence of high-risk HPV infection and the site preference of head and neck SCC. Nevertheless, because mouse and human keratinocytes may not respond exactly the same to HPV infection and the mouse model has limitations to recapitulate human oropharyngeal HPV oncogenesis, current results need to be interpreted with caution while more studies will be needed to explore the link between HPV-host-bacteria and the development of HPV-positive oropharyngeal cancer.

## References

[1] Gheit T. (2019). Mucosal and cutaneous human papillomavirus infections and cancer biology. Front Oncol.

[2] Scheffner M., Werness B.A., Huibregtse J.M., Levine A.J., Howley P.M. (1990). The E6 oncoprotein encoded by human papillomavirus types 16 and 18 promotes the degradation of p53. Cell.

[3] Scheffner M., Munger K., Huibregtse J.M., Howley P.M. (1992). Targeted degradation of the retinoblastoma protein by human papillomavirus E7-E6 fusion proteins. EMBO J.

[4] Duensing S., Lee L.Y., Duensing A., Basile J., Piboonniyom S., Gonzalez S., Crum C.P., Munger K. (2000). The human papillomavirus type 16 E6 and E7 oncoproteins cooperate to induce mitotic defects and genomic instability by uncoupling centrosome duplication from the cell division cycle. Proc Natl Acad Sci U S A.

[5] White A.E., Livanos E.M., Tlsty T.D. (1994). Differential disruption of genomic integrity and cell cycle regulation in normal human fibroblasts by the HPV oncoproteins. Genes Dev.

[6] DeFilippis R.A., Goodwin E.C., Wu L., DiMaio D. (2003). Endogenous human papillomavirus E6 and E7 proteins differentially regulate proliferation, senescence, and apoptosis in HeLa cervical carcinoma cells. J Virol.

[7] Horner S.M., DeFilippis R.A., Manuelidis L., DiMaio D. (2004). Repression of the human papillomavirus E6 gene initiates p53-dependent, telomerase-independent senescence and apoptosis in HeLa cervical carcinoma cells. J Virol.

[8] Saraiya M., Unger E.R., Thompson T.D., Lynch C.F., Hernandez B.Y., Lyu C.W., Steinau M., Watson M., Wilkinson E.J., Hopenhayn C., Copeland G., Cozen W., Peters E.S., Huang Y., Saber M.S., Altekruse S., Goodman M.T., HPV Typing of Cancers Workgroup (2015). US assessment of HPV types in cancers: implications for current and 9-valent HPV vaccines. J Natl Cancer Inst.

[9] Rietbergen M.M., van Bokhoven A., Lissenberg-Witte B.I., Heideman D.A.M., Leemans C.R., Brakenhoff R.H., Bloemena E. (2018). Epidemiologic associations of HPV-positive oropharyngeal cancer and (pre)cancerous cervical lesions. Int J Cancer.

[10] Brandsma J.L., Abramson A.L. (1989). Association of papillomavirus with cancers of the head and neck. Arch Otolaryngol Head Neck Surg.

[11] Lewis J.S., Beadle B., Bishop J.A., Chernock R.D., Colasacco C., Lacchetti C., Moncur J.T., Rocco J.W., Schwartz M.R., Seethala R.R., Thomas N.E., Westra W.H., Faquin W.C. (2018). Human papillomavirus testing in head and neck carcinomas: guideline from the College of American Pathologists. Arch Pathol Lab Med.

[12] Gillison M.L., Koch W.M., Capone R.B., Spafford M., Westra W.H., Wu L., Zahurak M.L., Daniel R.W., Viglione M., Symer D.E., Shah K.V., Sidransky D. (2000). Evidence for a causal association between human papillomavirus and a subset of head and neck cancers. J Natl Cancer Inst.

[13] Chernock R.D., Lewis J.S., Zhang Q., El-Mofty S.K. (2010). Human papillomavirus-positive basaloid squamous cell carcinomas of the upper aerodigestive tract: a distinct clinicopathologic and molecular subtype of basaloid squamous cell carcinoma. Hum Pathol.

[14] Miles B.A. (2019). Recent changes in the American Joint Commission Cancer staging of human papilloma virus related oropharyngeal cancer: validating the present and envisioning the future. Chin Clin Oncol.

[15] Gillison M.L., Trotti A.M., Harris J., Eisbruch A., Harari P.M., Adelstein D.J., Jordan R.C.K., Zhao W., Sturgis E.M., Burtness B., Ridge J.A., Ringash J., Galvin J., Yao M., Koyfman S.A., Blakaj D.M., Razaq M.A., Colevas A.D., Beitler J.J., Jones C.U., Dunlap N.E., Seaward S.A., Spencer S., Galloway T.J., Phan J., Dignam J.J., Le Q.T. (2019). Radiotherapy plus cetuximab or cisplatin in human papillomavirus-positive oropharyngeal cancer (NRG Oncology RTOG 1016): a randomised, multicentre, non-inferiority trial. Lancet.

[16] Mehanna H., Robinson M., Hartley A., Kong A., Foran B., Fulton-Lieuw T., Dalby M., Mistry P., Sen M., O'Toole L., Al Booz H., Dyker K., Moleron R., Whitaker S., Brennan S., Cook A., Griffin M., Aynsley E., Rolles M., De Winton E., Chan A., Srinivasan D., Nixon I., Grumett J., Leemans C.R., Buter J., Henderson J., Harrington K., McConkey C., Gray A., Dunn J., De-ESCALaTE HPV Trial Group (2019). Radiotherapy plus cisplatin or cetuximab in low-risk human papillomavirus-positive oropharyngeal cancer (De-ESCALaTE HPV): an open-label randomised controlled phase 3 trial. Lancet.

[17] Stanley M.A., Browne H.M., Appleby M., Minson A.C. (1989). Properties of a non-tumorigenic human cervical keratinocyte cell line. Int J Cancer.

[18] Pett M.R., Alazawi W.O., Roberts I., Dowen S., Smith D.I., Stanley M.A., Coleman N. (2004). Acquisition of high-level chromosomal instability is associated with integration of human papillomavirus type 16 in cervical keratinocytes. Cancer Res.

[19] Gray E., Pett M.R., Ward D., Winder D.M., Stanley M.A., Roberts I., Scarpini C.G., Coleman N. (2010). In vitro progression of human papillomavirus 16 episome-associated cervical neoplasia displays fundamental similarities to integrant-associated carcinogenesis. Cancer Res.

[20] Leemans C.R., Snijders P.J.F., Brakenhoff R.H. (2018). The molecular landscape of head and neck cancer. Nat Rev Cancer.

[21] Lerman M.A., Almazrooa S., Lindeman N., Hall D., Villa A., Woo S.B. (2017). HPV-16 in a distinct subset of oral epithelial dysplasia. Mod Pathol.

[22] Bishop J.A., Ma X.J., Wang H., Luo Y., Illei P.B., Begum S., Taube J.M., Koch W.M., Westra W.H. (2012). Detection of transcriptionally active high-risk HPV in patients with head and neck squamous cell carcinoma as visualized by a novel E6/E7 mRNA in situ hybridization method. Am J Surg Pathol.

[23] Perry M.E. (1994). The specialised structure of crypt epithelium in the human palatine tonsil and its functional significance. J Anat.

[24] Jensen A., Fago-Olsen H., Sorensen C.H., Kilian M. (2013). Molecular mapping to species level of the tonsillar crypt microbiota associated with health and recurrent tonsillitis. PLoS One.

[25] Segata N., Haake S.K., Mannon P., Lemon K.P., Waldron L., Gevers D., Huttenhower C., Izard J. (2012). Composition of the adult digestive tract bacterial microbiome based on seven mouth surfaces, tonsils, throat and stool samples. Genome Biol.

[26] Porter S.R., Scully C. (2006). Oral malodour (halitosis). BMJ.

[27] O'Keefe S.J. (2016). Diet, microorganisms and their metabolites, and colon cancer. Nat Rev Gastroenterol Hepatol.

[28] Pryde S.E., Duncan S.H., Hold G.L., Stewart C.S., Flint H.J. (2002). The microbiology of butyrate formation in the human colon. FEMS Microbiol Lett.

[29] Decrion-Barthod A.Z., Bosset M., Plissonnier M.L., Marchini A., Nicolier M., Launay S., Pretet J.L., Rommelaere J., Mougin C. (2010). Sodium butyrate with UCN-01 has marked antitumour activity against cervical cancer cells. Anticancer Res.

[30] Shen Z., Shen J., Cai W., Chen M., Wu X., Zheng R., Zeng Y. (2002). The promoter effects of sodium butyrate on the malignant transformation of the immortalized esophageal epithelium induced by human papillomavirus. Zhonghua Bing Li Xue Za Zhi.

[31] Carper M.B., Troutman S., Wagner B.L., Byrd K.M., Selitsky S.R., Parag-Sharma K., Henry E.C., Li W., Parker J.S., Montgomery S.A., Cleveland J.L., Williams S.E., Kissil J.L., Hayes D.N., Amelio A.L. (2019). An immunocompetent mouse model of HPV16(+) head and neck squamous cell carcinoma. Cell Rep.

[32] Ho G.Y., Bierman R., Beardsley L., Chang C.J., Burk R.D. (1998). Natural history of cervicovaginal papillomavirus infection in young women. N Engl J Med.

[33] Franco E.L., Villa L.L., Sobrinho J.P., Prado J.M., Rousseau M.C., Desy M., Rohan T.E. (1999). Epidemiology of acquisition and clearance of cervical human papillomavirus infection in women from a high-risk area for cervical cancer. J Infect Dis.

[34] Shanmugasundaram S., You J. (2017). Targeting persistent human papillomavirus infection. Viruses.

[35] Committee for the Update of the Guide for the Care and Use of Laboratory Animals (2011). Guide for the Care and Use of Laboratory Animals.

[36] Counter C.M., Hahn W.C., Wei W., Caddle S.D., Beijersbergen R.L., Lansdorp P.M., Sedivy J.M., Weinberg R.A. (1998). Dissociation among in vitro telomerase activity, telomere maintenance, and cellular immortalization. Proc Natl Acad Sci U S A.

[37] Woodworth C.D., Bowden P.E., Doniger J., Pirisi L., Barnes W., Lancaster W.D., DiPaolo J.A. (1988). Characterization of normal human exocervical epithelial cells immortalized in vitro by papillomavirus types 16 and 18 DNA. Cancer Res.

[38] Lu S.Y., Li M., Lin Y.L. (2014). Mitf regulates osteoclastogenesis by modulating NFATc1 activity. Exp Cell Res.

[39] Natarajan E., Saeb M., Crum C.P., Woo S.B., McKee P.H., Rheinwald J.G. (2003). Co-expression of p16(INK4A) and laminin 5 gamma2 by microinvasive and superficial squamous cell carcinomas in vivo and by migrating wound and senescent keratinocytes in culture. Am J Pathol.

[40] Bertolero F., Kaighn M.E., Camalier R.F., Saffiotti U. (1986). Effects of serum and serum-derived factors on growth and differentiation of mouse keratinocytes. In Vitro Cell Dev Biol.

[41] Hennings H., Michael D., Cheng C., Steinert P., Holbrook K., Yuspa S.H. (1980). Calcium regulation of growth and differentiation of mouse epidermal cells in culture. Cell.

[42] Liang C.C., Park A.Y., Guan J.L. (2007). In vitro scratch assay: a convenient and inexpensive method for analysis of cell migration in vitro. Nat Protoc.

[43] Bankhead P., Loughrey M.B., Fernandez J.A., Dombrowski Y., McArt D.G., Dunne P.D., McQuaid S., Gray R.T., Murray L.J., Coleman H.G., James J.A., Salto-Tellez M., Hamilton P.W., QuPath (2017). open source software for digital pathology image analysis. Sci Rep.

[44] Chen J.J. (2010). Genomic instability induced by human papillomavirus oncogenes. N Am J Med Sci (Boston).

[45] Webster M., Witkin K.L., Cohen-Fix O. (2009). Sizing up the nucleus: nuclear shape, size and nuclear-envelope assembly. J Cell Sci.

[46] Oda D., Bigler L., Mao E.J., Disteche C.M. (1996). Chromosomal abnormalities in HPV-16-immortalized oral epithelial cells. Carcinogenesis.

[47] de Haan J.B., Gevers W., Parker M.I. (1986). Effects of sodium butyrate on the synthesis and methylation of DNA in normal cells and their transformed counterparts. Cancer Res.

[48] Singh N.P., Lai H.C. (2005). Synergistic cytotoxicity of artemisinin and sodium butyrate on human cancer cells. Anticancer Res.

[49] Cancer Genome Atlas N. (2015). Comprehensive genomic characterization of head and neck squamous cell carcinomas. Nature.

[50] Pickering C.R., Zhang J., Yoo S.Y., Bengtsson L., Moorthy S., Neskey D.M., Zhao M., Ortega Alves M.V., Chang K., Drummond J., Cortez E., Xie T.X., Zhang D., Chung W., Issa J.P., Zweidler-McKay P.A., Wu X., El-Naggar A.K., Weinstein J.N., Wang J., Muzny D.M., Gibbs R.A., Wheeler D.A., Myers J.N., Frederick M.J. (2013). Integrative genomic characterization of oral squamous cell carcinoma identifies frequent somatic drivers. Cancer Discov.

[51] Del Bufalo D., Rizzo A., Trisciuoglio D., Cardinali G., Torrisi M.R., Zangemeister-Wittke U., Zupi G., Biroccio A. (2005). Involvement of hTERT in apoptosis induced by interference with Bcl-2 expression and function. Cell Death Differ.

[52] Yuan H., Fu F., Zhuo J., Wang W., Nishitani J., An D.S., Chen I.S., Liu X. (2005). Human papillomavirus type 16 E6 and E7 oncoproteins upregulate c-IAP2 gene expression and confer resistance to apoptosis. Oncogene.

[53] Marullo R., Werner E., Zhang H., Chen G.Z., Shin D.M., Doetsch P.W. (2015). HPV16 E6 and E7 proteins induce a chronic oxidative stress response via NOX2 that causes genomic instability and increased susceptibility to DNA damage in head and neck cancer cells. Carcinogenesis.

[54] Georgescu S.R., Mitran C.I., Mitran M.I., Caruntu C., Sarbu M.I., Matei C., Nicolae I., Tocut S.M., Popa M.I., Tampa M. (2018). New insights in the pathogenesis of HPV infection and the associated carcinogenic processes: the role of chronic inflammation and oxidative stress. J Immunol Res.

[55] Kroesen M., Gielen P., Brok I.C., Armandari I., Hoogerbrugge P.M., Adema G.J. (2014). HDAC inhibitors and immunotherapy; a double edged sword?. Oncotarget.

[56] Zhou C., Tuong Z.K., Frazer I.H. (2019). Papillomavirus immune evasion strategies target the infected cell and the local immune system. Front Oncol.

[57] Gillison M.L., Akagi K., Xiao W., Jiang B., Pickard R.K.L., Li J., Swanson B.J., Agrawal A.D., Zucker M., Stache-Crain B., Emde A.K., Geiger H.M., Robine N., Coombes K.R., Symer D.E. (2019). Human papillomavirus and the landscape of secondary genetic alterations in oral cancers. Genome Res.

[58] Cohen N., Gupta M., Doerwald-Munoz L., Jang D., Young J.E., Archibald S., Jackson B., Lee J., Chernesky M. (2017). Developing a new diagnostic algorithm for human papilloma virus associated oropharyngeal carcinoma: an investigation of HPV DNA assays. J Otolaryngol Head Neck Surg.

[59] Sarkar S., Abujamra A.L., Loew J.E., Forman L.W., Perrine S.P., Faller D.V. (2011). Histone deacetylase inhibitors reverse CpG methylation by regulating DNMT1 through ERK signaling. Anticancer Res.

[60] Handy D.E., Castro R., Loscalzo J. (2011). Epigenetic modifications: basic mechanisms and role in cardiovascular disease. Circulation.

[61] Eckschlager T., Plch J., Stiborova M., Hrabeta J. (2017). Histone deacetylase inhibitors as anticancer drugs. Int J Mol Sci.

[62] Ferreira A.R., Ramalho A.C., Marques M., Ribeiro D. (2020). The interplay between antiviral signaling and carcinogenesis in human papillomavirus infections. Cancers (Basel).

[63] Thompson L.D., Bishop J.A. (2019).

[64] Neville B.W.D., Douglas D., Allen C.M., Chi A.C. (2016).

